# Mild Head Trauma: Is Antiplatelet Therapy a Risk Factor for Hemorrhagic Complications?

**DOI:** 10.3390/medicina57040357

**Published:** 2021-04-07

**Authors:** Gabriele Savioli, Iride Francesca Ceresa, Sabino Luzzi, Alice Giotta Lucifero, Maria Serena Pioli Di Marco, Federica Manzoni, Lorenzo Preda, Giovanni Ricevuti, Maria Antonietta Bressan

**Affiliations:** 1 Emergency Department, Fondazione IRCCS Policlinico San Matteo, 27100 Pavia, Italy; irideceresa@gmail.com (I.F.C.); mspioli@smatteo.pv.it (M.S.P.D.M.); mita.bressan@gmail.com (M.A.B.); 2 PhD School in Experimental Medicine, Department of Clinical-Surgical, Diagnostic and Pediatric Sciences, University of Pavia, 27100 Pavia, Italy; 3 Neurosurgery Unit, Department of Clinical-Surgical, Diagnostic and Pediatric Sciences, University of Pavia, 27100 Pavia, Italy; sabino.luzzi@gmail.com (S.L.); alicelucifero@gmail.com (A.G.L.); 4 Neurosurgery Unit, Department of Surgical Sciences, Fondazione IRCCS Policlinico San Matteo, 27100 Pavia, Italy; 5 Health Promotion—Environmental Epidemiology Unit, Hygiene and Health Prevention Department, Health Protection Agency, 27100 Pavia, Italy; fmanzoni@smatteo.pv.it; 6 Radiology Unit, Fondazione IRCCS Policlinico San Matteo, 27100 Pavia, Italy; lpreda@smatteo.pv.it; 7 Department of Drug Science, University of Pavia, 27100 Pavia, Italy; giovanni.ricevuti@unipv.it; 8 Saint Camillus International University of Health Sciences, 00152 Rome, Italy

**Keywords:** minor head injury, mild head trauma, antiplatelet therapy, emergency department, bleeding risk, intracranial hemorrhage, cranial neurosurgical interventions, hospital admission

## Abstract

*Background and objectives:* In patients who receive antiplatelet therapy (APT), the bleeding risk profile after mild head trauma (MHT) still needs clarification. Some studies have demonstrated an association with bleeding risk, whereas others have not. We studied the population of our level II emergency department (ED) trauma center to determine the risk of bleeding in patients receiving APT and whether bleeding results not from antiplatelet agents but rather from age. We assessed the bleeding risk, the incidence of intracranial hemorrhage (ICH) that necessitated hospitalization for observation, the need for cranial neurosurgery, the severity of the patient’s condition at discharge, and the frequency of ED revisits for head trauma in patients receiving APT. *Materials and Methods:* This retrospective single-center study included 483 patients receiving APT who were in the ED for MHT in 2019. The control group consisted of 1443 patients in the ED with MHT over the same period who were not receiving APT or anticoagulant therapy. Our ED diagnostic therapeutic protocol mandates both triage and the medical examination to identify patients with MHT who are taking any anticoagulant or APT. *Results:* APT was not significantly associated with bleeding risk (*p* > 0.05); as a risk factor, age was significantly associated with the risk of bleeding, even after adjustment for therapy. Patients receiving APT had a greater need of surgery (1.2% vs. 0.4%; *p* < 0.0001) and a higher rate of hospitalization (52.9% vs. 37.4%; *p* < 0.0001), and their clinical condition was more severe (evaluated according to the exit code value on a one-dimensional quantitative five-point numerical scale) at the time of discharge (*p* = 0.013). The frequency of ED revisits due to head trauma did not differ between the two groups. *Conclusions:* The risk of bleeding in patients receiving APT who had MHT was no higher than that in the control group. However, the clinical condition of patients receiving APT, including hospital admission for ICH monitoring and cranial neurosurgical interventions, was more severe.

## 1. Introduction

Minor head injury (MHI), in which affected patients have a Glasgow Coma Scale score of 14 or 15, is among the most common reasons why people go to the emergency department (ED). MHI accounts for approximately 88% of cases of head trauma. Despite the low risk for severe complications, MHI carries a potential risk of a poor outcome [1]. For approximately 95% of patients with MHI, computed tomographic (CT) scans indicate no abnormality, and fewer than 1% are candidates for surgery. An increasing number of investigations are being conducted to identify the risk factors for intracranial hemorrhages [2,3,4,5,6,7,8,9,10].

Although anticoagulant therapy is recognized as a risk factor for bleeding, ongoing use of antiplatelet drugs is still controversial. Reports suggest that people receiving antiplatelet therapy tend to bleed, and whether this represents an independent risk factor for hemorrhagic complications is unclear. The tendency to bleed is more pronounced with age [11,12,13,14,15,16,17,18,19]. Since 2015, antiplatelets have increasingly been used as the primary method of preventing cardiovascular diseases [20,21,22,23].

The goal of our study was to assess the risk of bleeding in patients receiving antiplatelet therapy who visited our ED because of MHI. The secondary endpoints were hospitalization necessary for observation and monitoring intracranial hemorrhage (ICH), the need for cranial neurosurgical interventions for ICH, the severity of patients’ condition at discharge, and the rate of ED revisits for late bleeding.

## 2. Methods

### 2.1. Study Design

We conducted a retrospective single-center observational study involving all patients admitted to our ED in 2019 because of MHI. The primary endpoint was the diagnosis of post-traumatic ICH during observation and within 30 days. Secondary endpoints were the rate of hospitalization for observation and monitoring of ICH, the rate of ED revisits, the severity of patients’ condition at discharge, and the need for cranial neurosurgery during the hospital stay.

### 2.2. Inclusion and Exclusion Criteria

The study group involved patients with MHI, with Glasgow Coma Scale scores ≥14, aged >18 years, and receiving antiplatelet therapy (APT). The control group included patients with the same demographic data but who were not receiving APT or anticoagulant therapy and did not report any history of clotting disorders. The exclusion criteria were suspected or diagnosed skull fracture and spontaneous ICH. Of the patients whose records we reviewed, 129 were not eligible.

### 2.3. Study Population

The terms for our database search were “cranial trauma,” “intracranial hemorrhage,” and “head trauma.” Each patient’s data, including clinical reports, medical diary, and laboratory and radiological information, were examined with the use of the PIESSE digital platform.

We collected demographic data and information about the etiology and dynamic of injury, waiting time, process time, length of stay, blood pressure, means of arrival, triage code, exit code, and hematochemical examination results for all enrolled patients. In the triage guidelines of the American College of Surgeons, Sasser et al. described the dynamic criteria for major trauma [24]: ejection from a vehicle, motorcyclist thrown from a vehicle, death in a vehicle, intrusion of an interior compartment of >30 cm, and falling from a height of >2 m (Supplementary Table S1). When none of these criteria was present, the dynamics of trauma were considered to be minor. 

**Figure 1:**
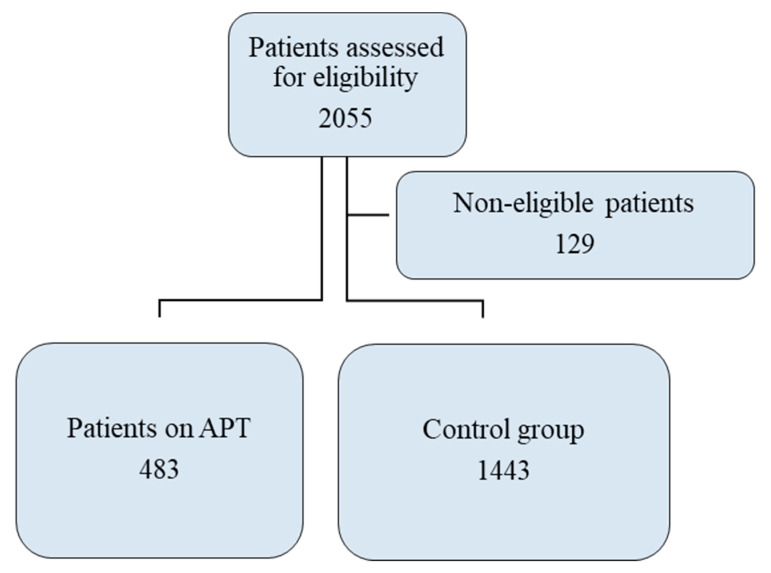
Study Flowchart.

Hospitalization for observation and monitoring of ICH, cranial surgery, death, and ED revisit within 30 days were documented. We viewed and evaluated all the performance data, and a neuroradiologist reviewed all CT scans. In patients receiving antiplatelet therapy, we reviewed each CT scan that was positive for ICH with regard to topography (intraparenchymal, subdural, epidural, and subarachnoid) and size. We also calculated the score on the Marshall classification of traumatic brain injury. Of the patients whose records we reviewed, 1926 consecutive patients with a diagnosis of MHI were enrolled; 483 were receiving APT and were assigned to the APT group, and the other 1443 were assigned to the control group (Figure 1).

A triage code was assigned according to predefined grids by the triage nurse to the patient. The exit code is a five-point quantitative one-dimensional numerical scale for assessing the severity of the patient according to medical judgment, scale in force in Italy. It was assigned by the emergency doctor to the patient at the time of admission based on clinical judgment. Exit code 1 represents the greatest severity, with 5 being the least.

In accordance with the International Emergency Severity Index and Canadian Triage and Acuity Scale, we used a modified system of triage coding involving five codes; code 1 represented the most severe condition of a patient upon entry into the ED, and code 5 represented the least severe condition (Table 1) [25,26,27,28,29,30,31,32,33,34,35,36,37]. At discharge, according to the clinical judgment of the ED physician, severity codes, called exit codes, that were based on the same scale as the triage codes (Table 1) were assigned to the patients. These were used to measure the severity of the patient’s condition at discharge.

### 2.4. Statistical Analysis

Continuous variables were calculated either as means ± standard deviations or as medians and interquartile ranges, depending on the normality of the distribution; categorical variables were calculated as counts and percentages.

Stata statistical software (Stata Corporation, College Station, TX, USA) was used. We used the *t* test to compare normally distributed data from the two groups; for other comparisons, we used the nonparametric Wilcoxon test. Associations between categorical variables were checked using Pearson’s χ^2^ test or Fisher’s exact test.

To study the association with the outcome variable (bleeding), we examined the potential covariates in the association between treatment and bleeding with univariable logistic models. After selecting the covariates (predictors for which a significant statistical association with the outcome variable bleeding had been reported), we tested logistic multivariable models with the treatment condition (APT vs. no APT) and each covariate as predictors.

To estimate the average treatment effect of APT versus no APT, we performed propensity score matching using a logistic model. The missing potential outcome for each patient receiving APT was determined from an average of the outcomes of similar patients who received APT, and that for each patient not receiving APT was determined according to similar patients not receiving APT. Similarity between patients was based on estimated treatment probabilities (propensity scores). To compute the treatment effect, we calculated the average of the differences between the observed and potential outcomes for each subject. Statistical significance was determined as *p* < 0.05, and all the tests were two-sided.

## 3. Results

Of the 1926 patients enrolled, 483 patients were receiving APT, and 1443 were not. Table 1 lists demographic and clinical data. Patients receiving APT had slightly lower heart rates, slightly lower oxygen saturation, and higher blood pressure. Scores on the Glasgow Coma Scale were similar in the two groups (*p* = 0.43). The APT group was, on average, older (approximately 81 vs. 54; *p* < 0.0001), and the cause of head injury in the group was more often minor (98.53 vs. 93.79%; *p* < 0.0001). The APT group had a greater number of high priority triage codes before the medical examination (*p* < 0.0001), shorter waiting times (approximately 46 vs. 72 min; *p* < 0.0001), and a longer length of stay in the ED (approximately 358 vs. 260 min; *p* < 0.0001; Table 1). Dynamic criteria for major trauma were present in only a small percentage of cases, for both the group on APT (1% (*n* = 5)) and the control group (4% (*n* = 58)). The causes of trauma are listed in Supplementary Table S2.

Among the APT group, 85% were taking acetylsalicylic acid; 9%, clopidogrel; 5%, ticlopidine; and 1%, indobuphen. APT did not appear to be statistically significant as a risk factor for bleeding, both when tested as the only predictor and after adjustment for age in the multivariable analysis. Age did appear to be a risk factor for bleeding, which was also true in the multivariable analysis, after adjustment for therapy (Table 2 and Table 3).

All the potential covariables in the association between treatment (APT vs. no APT) and the outcome (bleeding) were selected according to logistic regression models, as reported for statistical methodology. Diastolic blood pressure, trauma dynamics, oxygen saturation, and age ≥75 years were associated with bleeding, and these associations remained significant even after adjustment for treatment (APT vs. no APT; Table 4).

A logistic model with propensity score matching, based on the aforementioned covariables, was used to estimate the average treatment effect of APT versus no APT. As presented in Table 5, we found no statistically significant difference between the two groups after propensity score matching. Table 6 lists results of the univariable logistic model for the association between treatment (APT vs. no APT) and the outcome variable (bleeding) and the results for treatment after adjustment for each covariable with multivariable models.

With regard to secondary outcomes, the APT group had higher severity codes at discharge (*p* < 0.001), a higher rate of hospitalization (14.9% vs. 10.1%; *p* = 0.002; Table 1), more need of surgery (1.2% vs. 0.4%; *p* < 0.0001; Table 1), and a higher rate of ED revisits within 30 days of the injury for reasons related to head trauma, although this finding was not statistically significant (5.6% vs. 3.5%; *p* = 0.0608; Table 1). The various types of hemorrhage and the Marshall classifications of the patients are listed in Supplementary Table S3. Rates of hospital mortality did not differ between the groups.

## 4. Discussion

Our study did not demonstrate that APT was a risk factor for ICH. The findings indicate a higher rate of hospitalization to monitor ICH, a greater need for cranial surgery, and a worse condition at discharge among patients receiving APT.

The two groups analyzed differed mainly in age. It is possible that age also affected the other characteristics in which the two groups differed. Systolic blood pressure was higher in patients receiving APT, presumably because they are at greater cardiovascular risk and are older [38,39,40,41,42,43]. In fact, arterial hypertension is very common in patients harboring other cardiovascular risk factors [44,45]. Moreover, in patients receiving APT, falling was the most frequent mechanism of head trauma. In view of their having been older, falling could probably be related to age, as described in the literature [46,47,48].

The two groups were similarly hemodynamically stable. Glasgow Coma Scale scores were also similar. Inasmuch as MHI is defined on the basis of the Glasgow Coma Scale score, the similarity in scores highlights the comparability of the two groups.

The literature contains much debate as to whether APT predisposes to bleeding in the event of mild head trauma [1,12,49,50,51,52,53,54,55,56,57,58,59,60,61,62,63,64]. Our study indicates that APT is not related to a higher risk of bleeding, but it is related to a more severe clinical condition when bleeding does occur. In patients receiving APT, unfavorable conditions related to MHI, particularly the need for neurosurgery and hospitalization, are predictable. Patients receiving APT also need a longer stay in the ED, and longer lengths of stay are correlated with worse outcomes [57,65,66]. These patients, therefore, have complex problems, and during triage, it seems appropriate that they are classified as patients at greater risk.

Various results of studies have led to different protocols for adult and pediatric patients with MHI in the emergency room [11,12,13,14,15,16,17,18,19,67]. In some hospitals, all these patients undergo CT examination; in others, CT scanning is performed only if patients meet an age criterion (typically over 65 years) [2,3,4,5,6,7,8,20,21,22]. To our knowledge, however, different durations of observation have not been proposed. Thus, for patients who report MHI and are taking antiaggregation therapy, an observation period of 4–8 h is recommended [68]. Patients who are receiving APT, do not have focal deficits, and have stable lesions can be sent home after proper observation; patients receiving APT should be closely monitored, possibly for 24 h if they have bleeding.

Age remains instead an independent bleeding factor. Previous study results may have been skewed by the age variable. This possibility is supported by other studies [59,60,61,62,63,64,66,68,69]. Further studies are necessary to better delineate the risk profile of dual APT in patients with MHI.

### Limitations of the Study

This study had all the limitations of a retrospective and single-center study. The bleeding predictors analyzed were those that we deemed reliable in a retrospective study: dynamics of trauma, therapy in progress, Glasgow Coma Scale score, vital signs, sex, and age. We did not consider amnesia because it is not always clearly reported.

The rates of mortality in the two groups were not significantly different; however, we decided not to study it because the low incidence of death in cases of MHI would have necessitated a greater number of patients to be enrolled.

## 5. Conclusions

APT is not significantly associated with the rate of ICH in patients with MHI. However, such patients have a greater need for cranial neurosurgical intervention, longer hospitalization, and more frequent ED revisits.

## Figures and Tables

**Table 1:** Descriptive statistics for overall sample and distinctly per group.

Variable	Overall Sample (*n* = 1926)	Antiplatelet Therapy (APT) (*n* = 483)	Control Group (*n* = 1443)	*p*-Value
**Demographic and clinical data**				
Age, mean (SD)	60.71 (23.21)	80.67 (9.91)	54.02 (22.55)	<0.0001
Sex_ M, *n* (%)	941 (48.86)	208 (43.06)	733 (50.8)	0.004
Heart rate, mean (SD)	79.38 (13.52)	76.53 (12.39)	80.49 (13.78)	<0.0001
Breath frequency, mean (SD)	18.93 (5.69)	20.2 (9.67)	18.28 (1.25)	0.293
Oxygen saturation, mean (SD)	97.26 (2.37)	96.44 (2.52)	97.58 (2.23)	<0.0001
Diastolic blood pressure, mean (SD)	78.95 (12.83)	78.32 (13.76)	79.19 (12.45)	0.335
Systolic blood pressure, mean (SD)	139.85 (23.4)	147.77 (26)	136.76 (21.54)	<0.0001
Mean arterial pressure, mean (SD)	81.34 (40.37)	88.12 (37.37)	78.91 (41.14)	<0.001
Glasgow Coma Scale = 15, *n* (%)	1891(98.18)	472 (97.72)	1419 (98.34)	0.430
**Dynamics of trauma**				
Minor dynamics of trauma, *n* (%)	1800(94.99)	470 (98.53)	1330 (93.79)	<0.0001
**Triage codes**				
Code 1, *n* (%)	32(1.66)	4 (0.83)	28 (1.94)	<0.0001
Code 2, *n* (%)	692(35.93)	350 (72.46)	342 (23.7)	
Code 3, *n* (%)	33(1.71)	3 (0.62)	30 (2.08)	
Code 4, *n* (%)	1159(60.18)	126 (26.09)	1033 (71.59)	
Code 5, *n* (%)	10(0.52)	0 (0)	10 (0.69)	
**Management times**				
Time to doc, median (interquartile range)	62.7 (27.37; 121)	46.08 (23; 84.37)	71.43 (30.47; 132.03)	<0.0001
Length of ED stay, median (interquartile range)	285.2417 (176; 419.65)	357.6 (250.38; 531.38	260.28 (159.55;383.75)	<0.0001
**Exit code**				
Code 1, *n* (%)	12(0.68)	3 (0.64)	9 (0.69)	0.013
Code 2, *n* (%)	189(10.68)	67 (14.23)	122 (9.39)	
Code 3, *n* (%)	3(0.17)	0 (0)	3 (0.23)	
Code 4, *n* (%)	1550(87.57)	400 (84.93)	1150 (88.53)	
Code 5, *n* (%)	16(0.9)	1 (0.21)	15 (1.15)	
**Secondary outcomes**				
Emergency Department revisit in 30 days—yes, *n* (%)	78(4.05)	27 (5.59)	51 (3.53)	0.0608
Hospitalization, *n* (%)	218 (17.74)	72 (14.91)	146 (10.12)	0.002
Need of surgery, *n* (%)	12(0.62)	6 (1.24)	6 (0.41)	<0.0001

*p* values for the comparisons between the two groups are reported. Data are reported with mean and standard deviation or with median and interquartile range for continuous variables. Categorical variables are expressed with counts and percentages. Triage Code: 1 Lack of consciousness, highly unstable vital parameters. Need of immediate life-saving intervention; 2 = Unstable vital parameters; 3 = Stable vital parameters, but multiple resources need to stabilize the patient. Deferable urgency cases. 4 = Minor emergencies. One resource needed to stabilize the patient. 5 = non-urgent cases. No resources needed to stabilize the patient. Exit Code: 1 = Coma, shock, highly unstable hemodynamics or breath despite intensive cares in the emergency department; 2 = Unstable vital parameters; 3 = Stable vital parameters; 4 = Minor emergencies; 5 = Nonurgent cases.

**Table 2:** Univariate logistic models for bleeding risk.

	OR	IC 95% OR	*p*-Value
**Logistic Model 1 (Univariate analysis)—Predictor: Therapy (APT vs. no APT)**			
	1.39	0.96–2.01	0.085
**Logistic Model 2 (Univariate analysis)—Predictor: Therapy (age considered as a continuous variable)**			
	1.02	1.01–1.03	<0.001
**Logistic Model 3 (Univariate analysis)—Predictor: Therapy (age categorized: age ≥ 75 years vs. age < 75 years)**			
	2.38	1.66–3.41	<0.001

**Table 3:** Multivariate logistic models for bleeding risk.

**Logistic Model 4 (Multivariate Analysis)—Predictors: Therapy Adjusted for Age (Age Considered as a Continuous Variable)**			
**Predictor**	**OR**	**IC 95% OR**	***p*** **-Value**
**Therapy (APT vs. no APT)**	0.82	0.54–1.23	0.335
**Age (years)**	1.02	1.01–1.03	<0.001
**Logistic Model 4 (Multivariate Analysis)—Predictors: Therapy Adjusted for Age (Age Categorized: Age ≥ 75 Years vs. Age < 75 Years)**			
**Predictor**	**OR**	**IC 95% OR**	***p*** **-Value**
**Therapy (APT vs. no APT)**	0.87	0.58–1.33	0.528
**Age (≥75 years vs. <75 years)**	2.52	1.69–3.77	<0.001

**Table 4:** Logistic regression models for the association between covariates and bleeding outcome.

	Univariate Logistic Regression Model		Multivariate Logistic Regression Model (Adjusting for Treatment)	
Covariates	OR	*p* Value for the Association	OR	*p* Value for the Association
DBP	0.98	0.024	0.98	0.024
Trauma dynamics	2.42	0.002	2.71	0.001
O_2_ saturation	0.88	0.001	0.08	0.001
Age ≥ 75 years	2.37	<0.001	2.52	<0.001

DBP = diastolic blood pressure.

**Table 5:** Logistic regression model for the treatment-effects estimation with propensity score matching.

	OR	IC 95% OR	*p* Value
ATE (APT vs. no APT)	1.058	0.803; 1.399	0.686

**Table 6:** Logistic regression model for the for the association between treatment (APT vs. no APT) and bleeding outcome.

	OR	IC 95% OR	*p* Value
Univariate model			
APT (APT vs. no APT)	1.39	0.96; 2.01	0.085
Multivariate models adjusting for each covariate			
DBP	1.22	0.80; 1.87	0.360
Trauma dynamics	1.55	1.06; 2.27	0.023
O_2_ saturation	1.07	0.69; 1.65	0.770
Age ≥ 75 years	0.87	0.58; 1.33	0.528

## Data Availability

MDPI Research Data Policies.

## Supplementary Materials

The following are available online at https://www.mdpi.com/article/10.3390/medicina57040357/s1, Table S1. Triage criteria for severe trauma (one at least). Table S2. Causes of Mild Head Trauma. Table S3. Types of Bleedings.

## Abbreviations

APT	Antiplatelet Therapy
CT	Computed Tomography
ED	Emergency Department
GCS	Glasgow Coma Scale
ICH	Intracranial hemorrhage
LOS	Length of Stay
MHI	Minor Head Injuries
