# Supplementary material for: Mild Head Trauma: Is Antiplatelet Therapy a Risk Factor for Hemorrhagic Complications?
Source: Medicina (Kaunas). 2021 Apr 7;57(4):357. doi: 10.3390/medicina57040357 (PMC8067857; doi:10.3390/medicina57040357)
Supplement: Supplementary file 1 [file medicina-57-00357-s001.pdf]

Supplementary Materials

Table S1. Triage criteria for severe trauma (one at least).

Dynamic Criteria	Anatomical Criteria	Physiological Criteria
Eject from the vehicle	Penetrating head/neck/throat/abdomen/pelvic/armpit/groin trauma	Systolic blood pressure < 90 mmHg
Motorcyclist thrown from the vehicle	Amputations above the wrist or ankle	Respiratory or breathlessness rate <10 or >29 acts/min
Died in the same vehicle	Chest trauma with flap/costal volet	State of consciousness (GCS) <13
Intruding of the cockpit >30 cm	Neurological injury with paralysis of even a single limb	
Fall from height >2 m	Fractures of two or more subxinextising bones	

Table S2. Causes of Mild Head Trauma.

Cause of Trauma	APT (n. 483)	NO APT (n. 1443)
Falls—n. (%)	343 (71)	606 (42)
Accidental collision—n. (%)	48 (10)	260 (18)
Violence—n. (%)	5 (1)	115 (8)
Minor road—n. (%)	14 (3)	116 (8)
Syncope—n. (%)	43 (9)	101 (7)
Major road—n. (%)	5 (1)	58 (4)
Other—n. (%)	24 (5)	159 (11)

Table S3. Types of Bleedings.

Different Types of Bleedings	APT	NO APT
Subarachnoid hemorrhages—n. (%)	25 (5.2)	48 (3.3)
Intraparenchymal—n. (%)	17 (3.5)	14 (1)
Subdural—n. (%)	23 (4.8)	26 (1.8)
Epidural—n. (%)	2 (0.5)	36 (2.5)
